# Supplementary material for: Bursts from the past: Intrinsic properties link a network model to zebra finch song
Source: bioRxiv. 2025 Aug 4:2024.05.18.594825. Preprint. [Version 3] doi: 10.1101/2024.05.18.594825 (PMC11118566; doi:10.1101/2024.05.18.594825)
Supplement: Supplement 1 [file NIHPP2024.05.18.594825v3-supplement-1.pdf]

bioRxiv preprint doi: https://doi.org/10.1101/2024.05.18.594825; this version posted August 4, 2025. The copyright holder for this preprint (which was not certified by peer review) is the author/funder, who has granted bioRxiv a license to display the preprint in perpetuity. It is made available under aCC-BY 4.0 International license.

Supplemental Figures

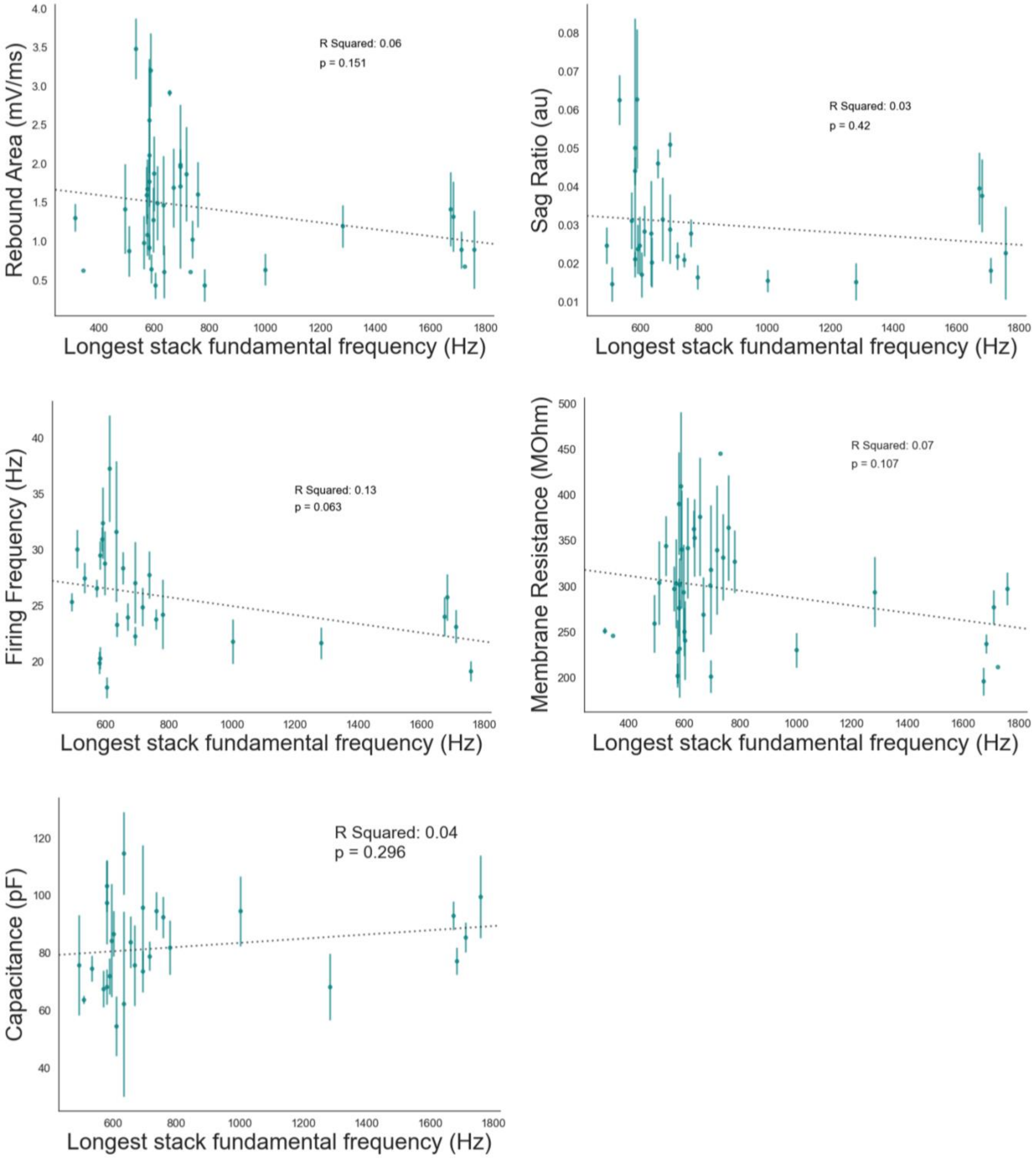

Supplemental Figure 1. Intrinsic properties are unrelated to fundamental frequency of longest harmonic stack. Scatter plots of mean analyzed parameters for all HVC_X for birds singing natural songs, against the fundamental frequency of the longest harmonic stack. Each point is the mean value for each bird, and error bars represent standard error of the mean.

bioRxiv preprint doi: https://doi.org/10.1101/2024.05.18.594825; this version posted August 4, 2025. The copyright holder for this preprint (which was not certified by peer review) is the author/funder, who has granted bioRxiv a license to display the preprint in perpetuity. It is made available under aCC-BY 4.0 International license.

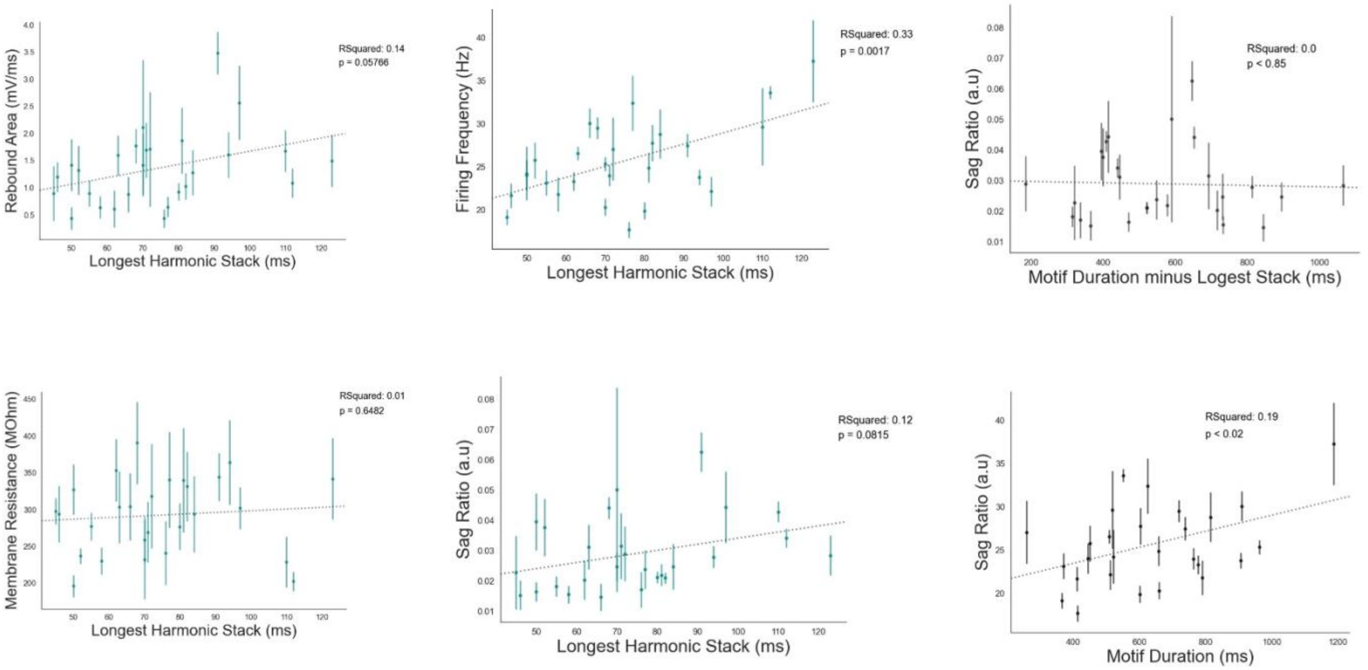

Supplemental Figure 2. Relationships between Intrinsic properties and song features, excluding birds whose songs had long-duration (> 150 ms) longest harmonic stacks. Scatter plots of mean analyzed parameters for all HVC neurons for each bird, against features of song duration (error bars are standard error of the mean).

bioRxiv preprint doi: https://doi.org/10.1101/2024.05.18.594825; this version posted August 4, 2025. The copyright holder for this preprint (which was not certified by peer review) is the author/funder, who has granted bioRxiv a license to display the preprint in perpetuity. It is made available under aCC-BY 4.0 International license.

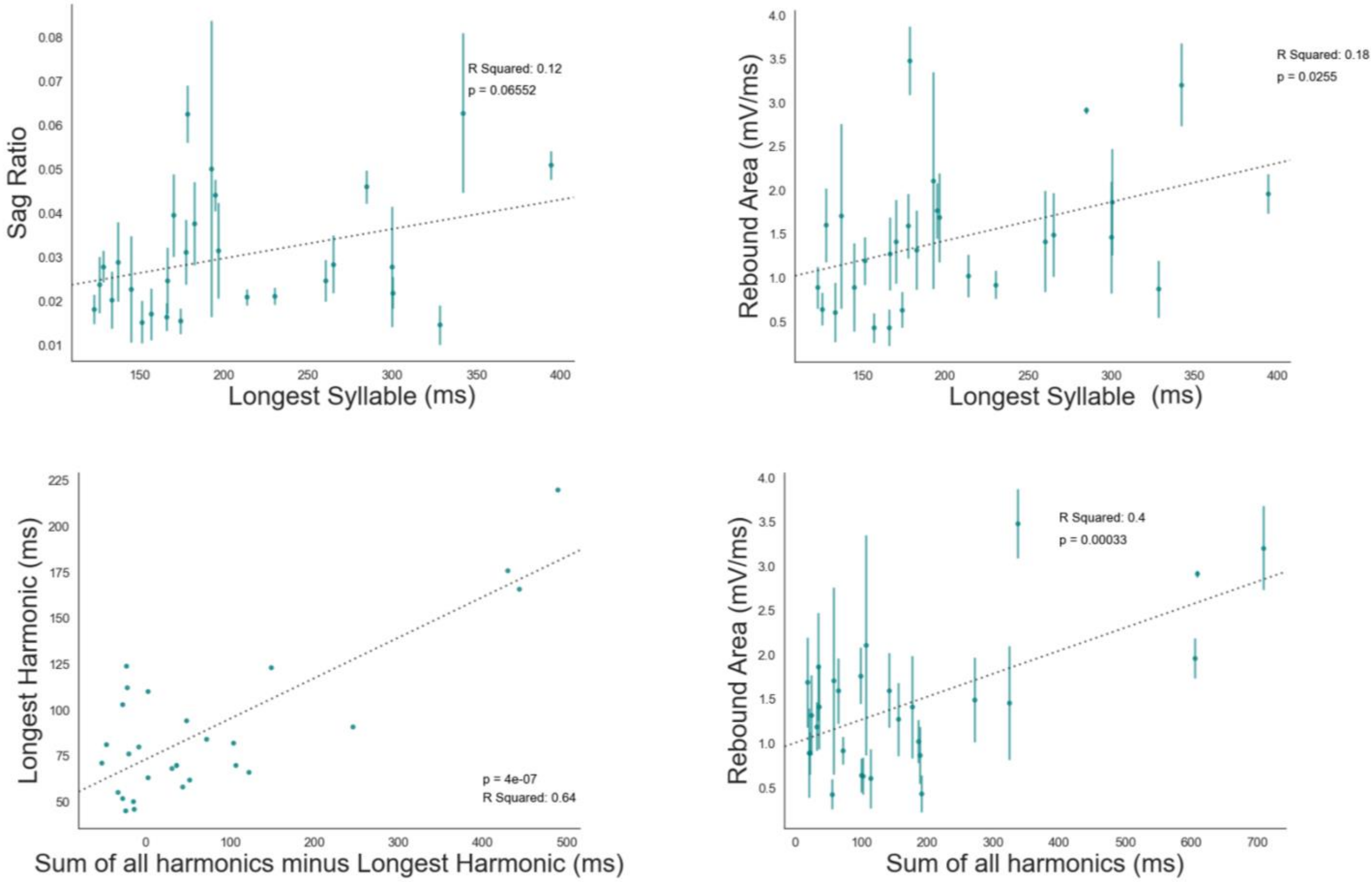

Supplemental Figure 3. Additional correlations between intrinsic properties and temporal song structure. Scatter plots of mean analyzed parameters for all HVC_X for birds singing natural songs, against longest syllable and the sum of additional harmonic elements beyond the longest harmonic. Error bars represent standard error of the mean.

bioRxiv preprint doi: https://doi.org/10.1101/2024.05.18.594825; this version posted August 4, 2025. The copyright holder for this preprint (which was not certified by peer review) is the author/funder, who has granted bioRxiv a license to display the preprint in perpetuity. It is made available under aCC-BY 4.0 International license.

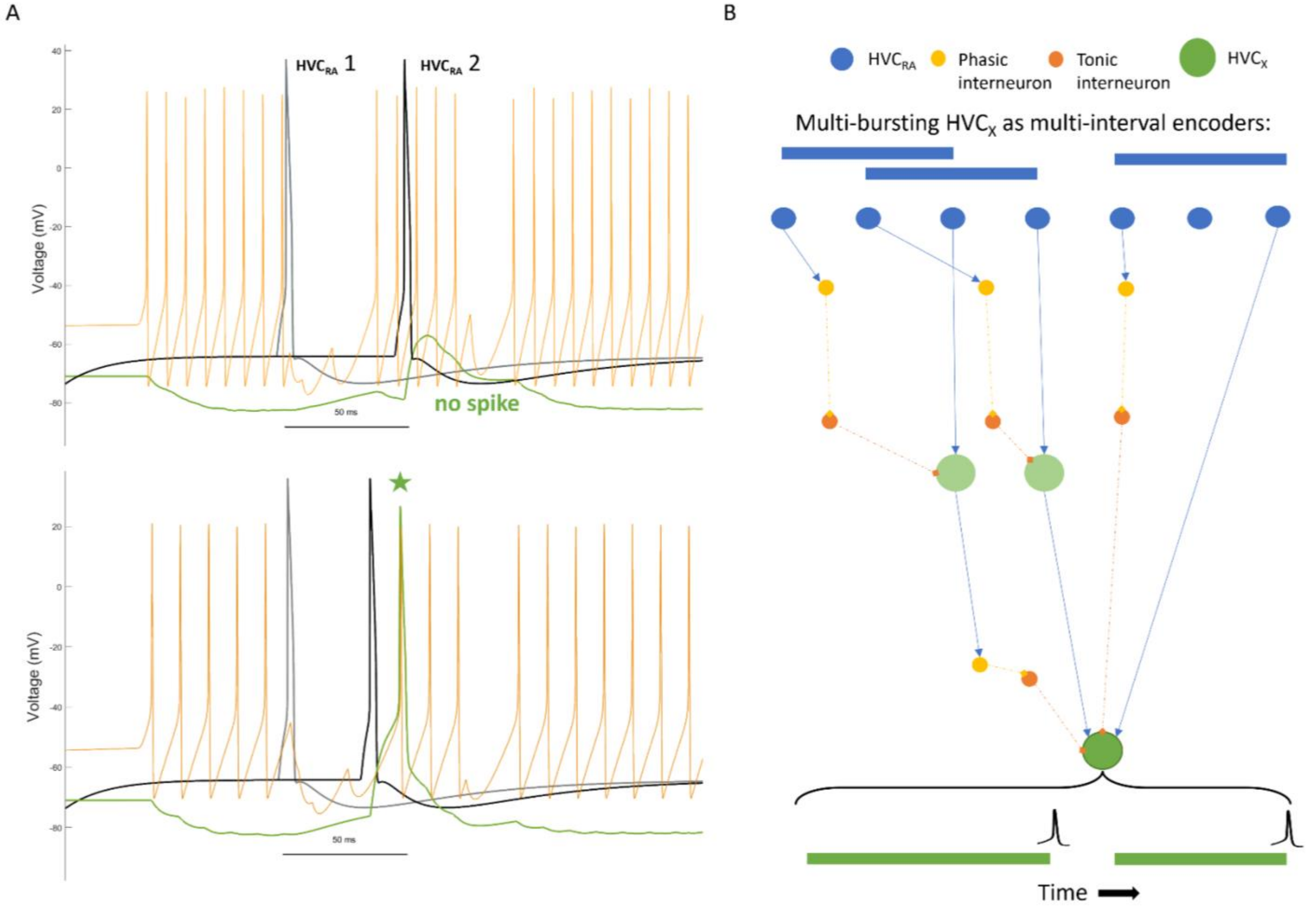

Supplemental Figure 4. Single and multi-bursting model HVC_X neurons. A: Voltage traces from multiple neurons modeled and wired as described in Figure 5, illustrating the time dependence for the sequence sensitivity of the network model. An interneuron (orange trace) inhibits an HVC_X (green trace). One HVC_{RA} (first grey spike) di-synaptically inhibits the orange interneuron while a second, later-bursting HVC_{RA} (later black spike) excites the green HVC_X neuron. The top panel shows the outcome where the second spike arrives too late, resulting in no spike in the HVC_X. The bottom panel shows a well-timed second HVC_{RA} spike producing a spike in the HVC_X (green star). **B:** Model circuit diagram depicting nested intervals leading to one HVC_X neuron (bottom dark green circle) that bursts twice.

bioRxiv preprint doi: https://doi.org/10.1101/2024.05.18.594825; this version posted August 4, 2025. The copyright holder for this preprint (which was not certified by peer review) is the author/funder, who has granted bioRxiv a license to display the preprint in perpetuity. It is made available under aCC-BY 4.0 International license.

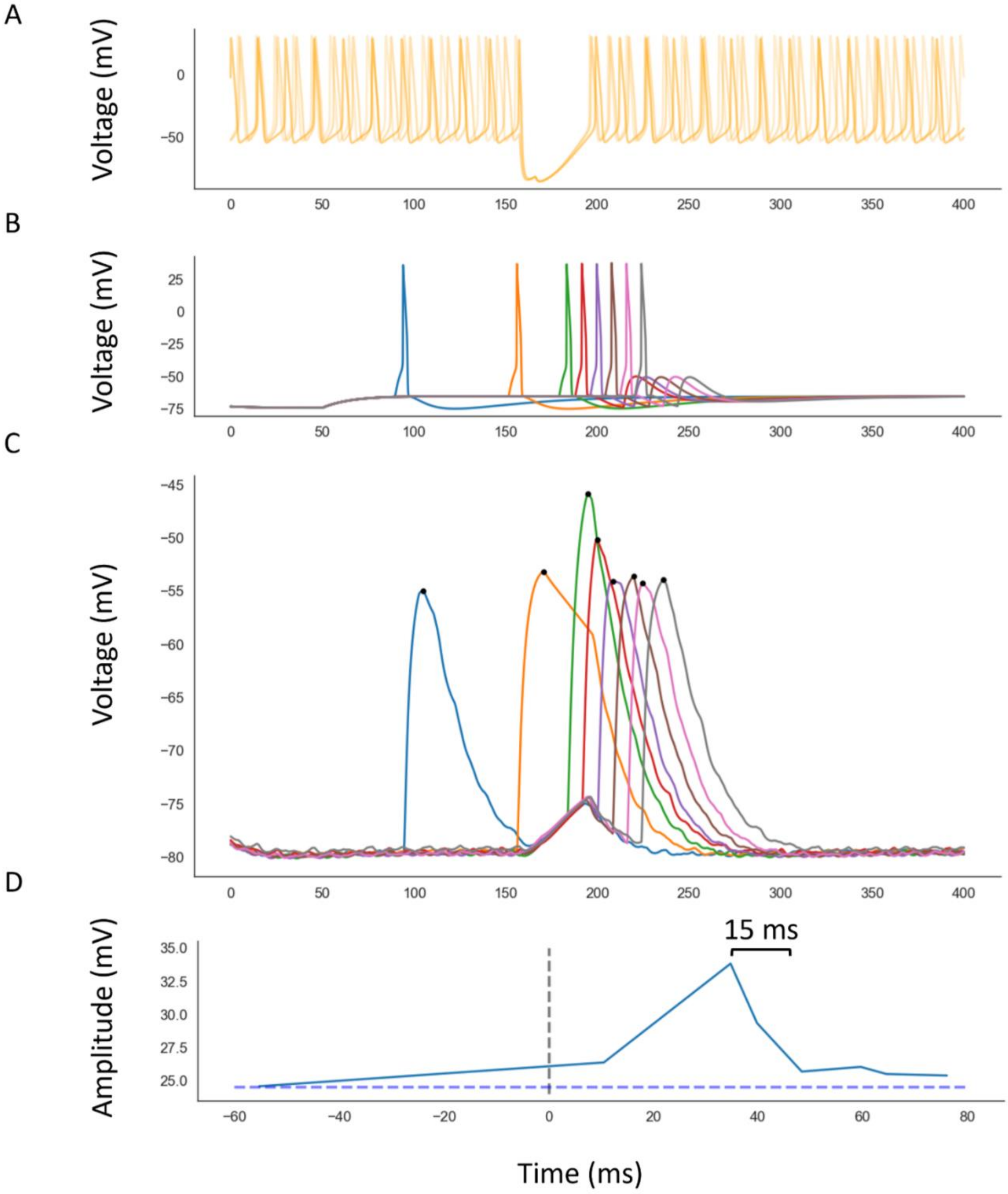

Supplemental Figure 5. Time window encoded by HVC$_X$. Hodgkin-Huxley model module with an HVC$_X$ neuron with no voltage-gated sodium channels and varying timing of excitatory inputs. **A:** Overlayed traces from three interneurons synapsing onto one HVC$_X$. **B:** Multiple HVC$_{RA}$ voltage traces overlayed (each HVC$_{RA}$ is depicted by a different color). **C:** The voltage traces of the same HVC$_X$ arising from inputs from interneurons in A, and each individual HVC$_{RA}$ in B. Peak voltages are shown by black dots. **D:** Peak amplitudes from C, and their relative timing from inhibition release (black dashed line). Baseline amplitude (blue dashed line) was taken from excitatory input that occurred before, and does not overlap with, release from inhibition.

bioRxiv preprint doi: https://doi.org/10.1101/2024.05.18.594825; this version posted August 4, 2025. The copyright holder for this preprint (which was not certified by peer review) is the author/funder, who has granted bioRxiv a license to display the preprint in perpetuity. It is made available under aCC-BY 4.0 International license.

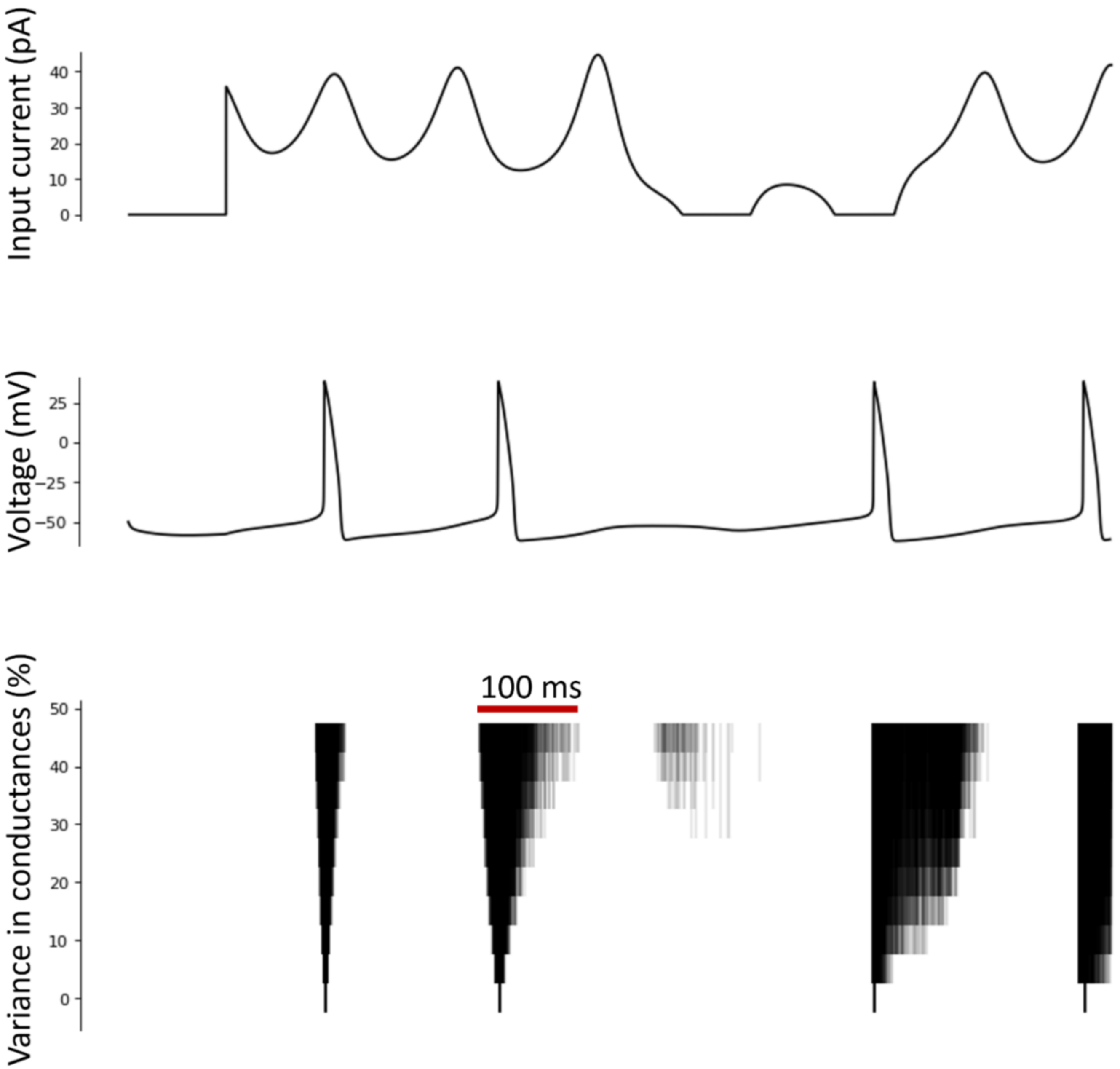

Supplemental Figure 6. Intrinsic property homogeneity promotes spike time homogeneity in modeled neurons. Hodgkin-Huxley model neurons receiving identical inputs (top panel) and producing differently timed spike responses (one example model trace, middle panel). Adjusting percent variance among five modeled ionic conductances (gNa, gK, gH, gSK, and gCa-T) between 0 and 50% produced different ranges spike times (bottom panel). Each row in the bottom panel represents all spike times for 100 modeled neurons at a given range of IP variance.

bioRxiv preprint doi: https://doi.org/10.1101/2024.05.18.594825; this version posted August 4, 2025. The copyright holder for this preprint (which was not certified by peer review) is the author/funder, who has granted bioRxiv a license to display the preprint in perpetuity. It is made available under aCC-BY 4.0 International license.

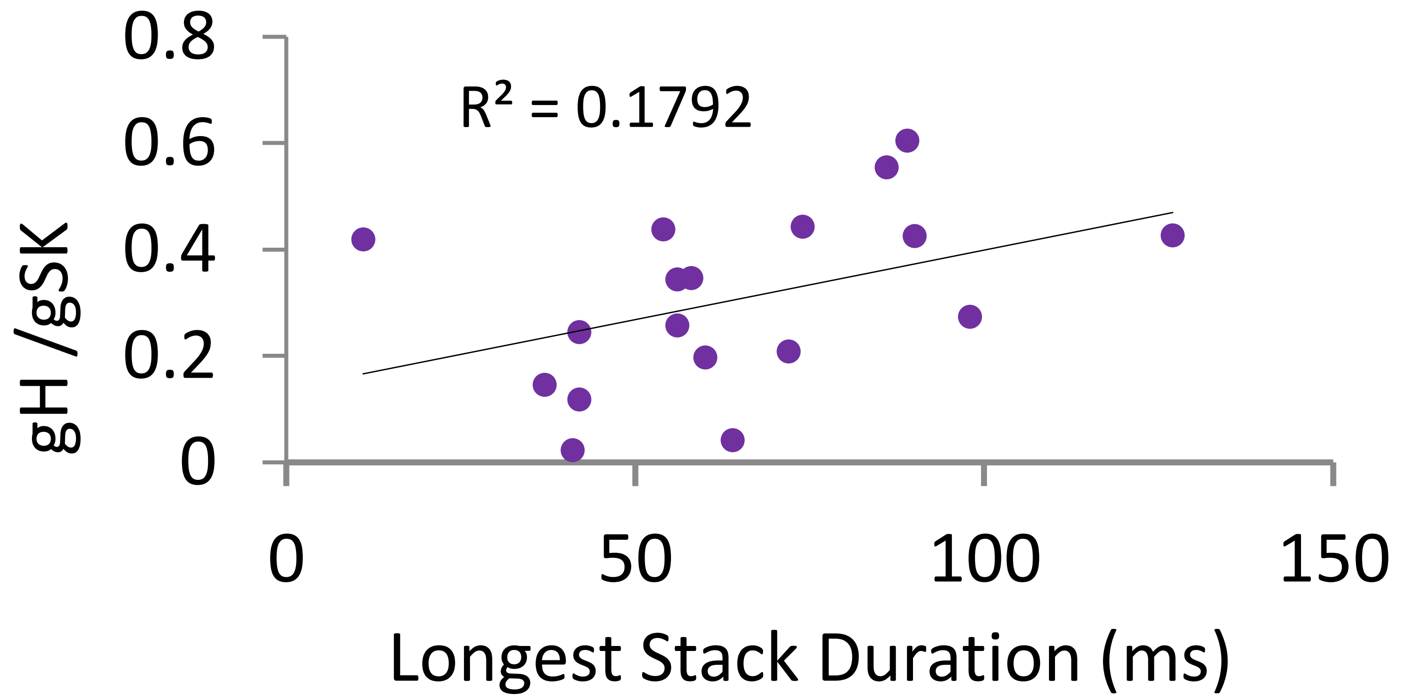

Supplemental Figure 7. Relationships between modeled conductances and longest harmonic from Daou and Margoliash 2020. Scatter plots of mean gH divided by gSH for all HVC neurons for 18 birds.
